# Review of Allergic and Photoallergic Contact Dermatitis from an Ingredient in a Medicament Vehicle Consisting of a Compress, Poultice, Plaster, and Tape

**DOI:** 10.1155/2011/169432

**Published:** 2011-04-06

**Authors:** Naoki Oiso, Akira Kawada

**Affiliations:** Department of Dermatology, Faculty of Medicine, Kinki University, 377-2 Ohno-Higashi, Osaka-Sayama, Osaka 589-8511, Japan

## Abstract

The topical application of a medicament vehicle consisting of a compress, poultice, plaster, and tape containing a nonsteroidal anti-inflammatory drug or methyl salicylate is prevalent in Japan. The method is effective for conveying ingredients to the muscles via the skin for the relief of muscular pain. However, an ingredient in the occlusive vehicle can cause allergic and photoallergic contact dermatitis. We summarize cases reported over the past decade and discuss the current strategy for diminishing the risk of allergic and photoallergic contact dermatitis.

## 1. Introduction

The application of a topical medicament consisting of a compress, poultice, plaster, and tape is prevalent in Japan. The occlusive vehicle is effective for conveying ingredients to the muscles via the skin. The vehicle usually contains a nonsteroidal anti-inflammatory drug (NSAID) or methyl salicylate as the effective component. It may also contain dl-camphor for relief of peripheral pain, l-menthol for peripheral cooling, and other ingredients, such as paraben, modified rosin, oxybenzone, and diisopropanolamine. We summarize cases of allergic and photoallergic contact dermatitis from an ingredient that were reported during the last decade [1–15].

## 2. Allergic and Photoallergic Contact Dermatitis

The occlusive application enhances the penetration of the effective substances. However, increased penetration may provoke allergic and photoallergic contact dermatitis from an ingredient. Allergic and photoallergic sensitization to two or more allergic or photoallergic substances can simultaneously occur [2, 6, 12]. Patch and photopatch testing with all of components is indispensable for precise diagnosis. 

The effective components, an NSAID [6] or methyl salicylate [8], have been shown to be allergens. Additives, such as crotamiton [6], diisopropanolamine [6, 10, 15], l-menthol [12, 14], paraben [7], and modified resin [11, 12] also have been shown to be allergens. Benzalkonium chloride usually induces irritant contact dermatitis, but rarely induces allergic contact dermatitis [5, 16–18]. 

 Ingredients such as ketoprofen [1–4, 12] and oxybenzone [2] have been shown to be photoallergens. The most hazardous is ketoprofen because of the highly frequent occurrence of photoallergic contact dermatitis [1–4, 12]. The mouse model of photoallergic contact dermatitis from ketoprofen has been established and the pathogenic mechanism has been investigated [19, 20].

**Figure 1:**
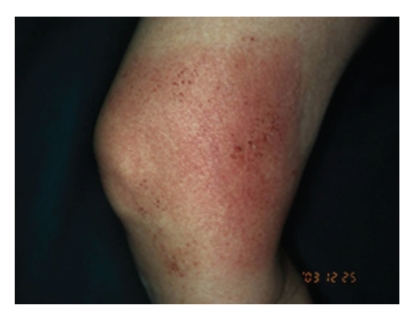
A 68-year-old Japanese woman with a rectangular pruritic erythematous macular area on the right knee.

**Figure 2:**
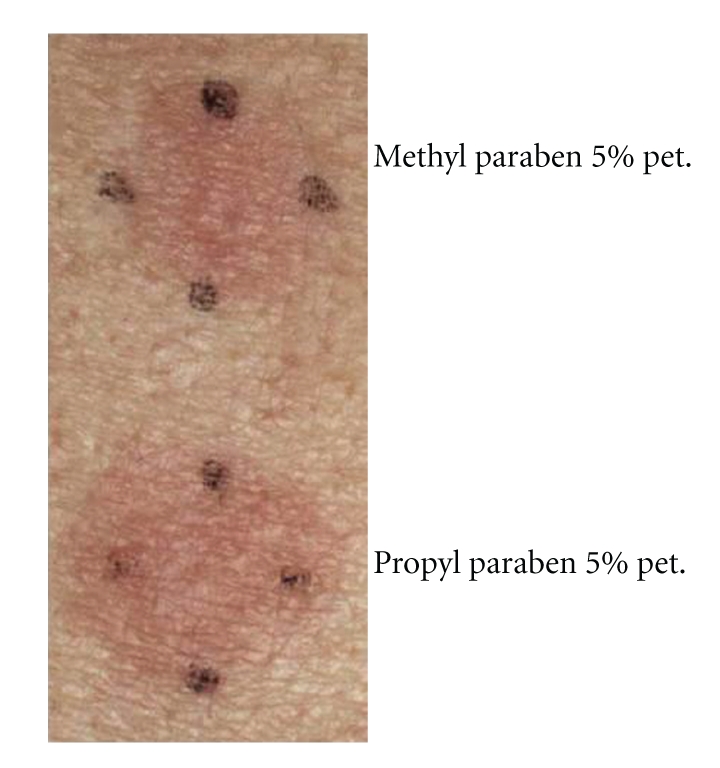
Patch testing for Case 1 showed positive reactions to methyl and propyl paraben at day 4.

The clinical feature is typically eczematous reactions, pruritic papular, vesicular, and bullous appearance. The size and shape are dictated by those of the applied vehicle, which is generally rectangular. Case 1 was a 68-year-old Japanese woman with a rectangular pruritic erythematous macular area on the right knee (Figure 1) [7]. In Case 1, patch testing showed a positive reaction at day 2 and 4 to the methyl and propyl paraben contained in the compress that had been used (Figure 2) [7]. 

**Figure 3:**
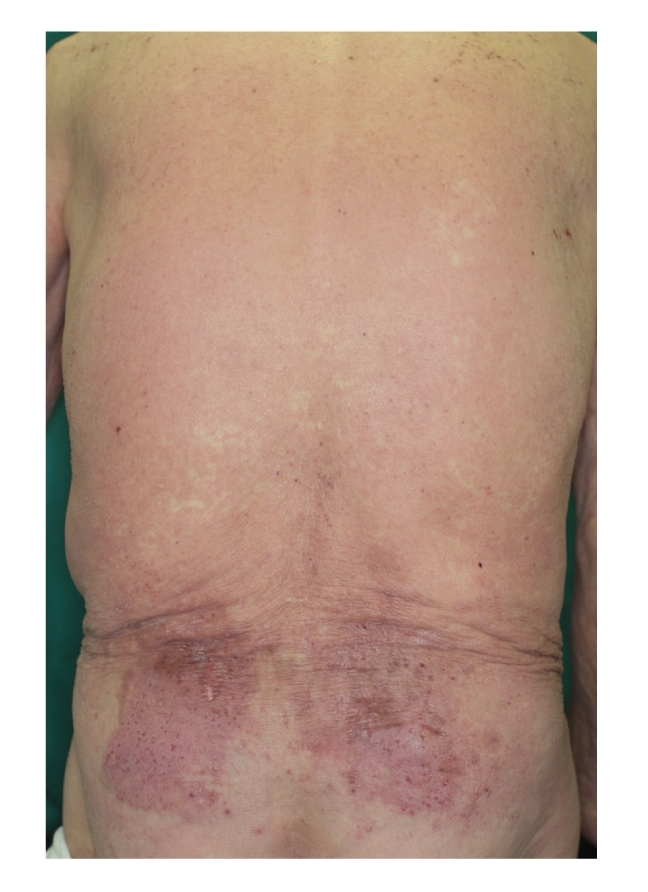
An 87-year-old Japanese male with rectangular erythema on the bilateral lower back and buttocks and a diffuse erythema on the trunk and extremities.

Some cases may show a rectangular eruption with a diffuse erythematous [6] or erythema multiform-like generalized reaction [14]. Case 2 was an 87-year-old Japanese male with a rectangular erythema on the bilateral lower back and the buttock and a diffuse erythema on the trunk and extremities caused by allergic contact dermatitis from the diisopropanolamine in the compresses that he used (Figure 3) [15]. 

Rectangular pruritic erythema may occur only when the lesion is exposed to sunlight. The effective component of the NSAID, such as ketoprofen, causes photoallergic contact dermatitis [1–4, 12, 13]. In such cases, a rectangular-shaped dermatitis with spreading [1] or erythema multiform-like eruption [13] is seen. Photoallergic contact dermatitis can be evoked by exposure to sunlight several weeks later after stopping the use of the occlusive products containing ketoprofen, because even several weeks after discontinuing the use of a poultice containing ketoproten, the skin still contains enough ketoprofen to trigger a reaction [1].

Strategies to diminish the risk of allergic and photoallergic contact dermatitis are promoted. One is the use of a topical cream, gel, or stick containing a low-sensitizing NSAID, such as felbinac [6] or loxoprofen. Another is the use of a topical occlusive medicament containing a low-sensitizing NSAID. However, physicians and pharmacologists must keep in mind that systemic contact and photocontact-type dermatitis may be evoked if a person previously sensitized to an NSAID orally takes the same NSAID [21]. 

In conclusion, the application of a vehicle consisting of a compress, poultice, plaster, and tape carries a greater risk of sensitization and elicitation of allergic and photoallergic contact dermatitis from an ingredient. For safety, we initially recommend the use of a topical cream, gel, or stick containing a less sensitizing ingredient, and secondarily a topical occlusive medicament containing a less sensitizing NSAID.
